# Development of a lung slice preparation for recording ion channel activity in alveolar epithelial type I cells

**DOI:** 10.1186/1465-9921-6-40

**Published:** 2005-04-27

**Authors:** Steven Bourke, Helen S Mason, Zea Borok, Kwang-Jin Kim, Edward D Crandall, Paul J Kemp

**Affiliations:** 1 Cardiff School of Biosciences, Museum Avenue, Cardiff CF10 3US, Wales, UK; 2 Will Rogers Institute Pulmonary Research Center, Division of Pulmonary and Critical Care Medicine, Keck School of Medicine, Los Angeles, CA 90033, USA

## Abstract

**Background:**

Lung fluid balance in the healthy lung is dependent upon finely regulated vectorial transport of ions across the alveolar epithelium. Classically, the cellular locus of the major ion transport processes has been widely accepted to be the alveolar type II cell. Although evidence is now emerging to suggest that the alveolar type I cell might significantly contribute to the overall ion and fluid homeostasis of the lung, direct assessment of functional ion channels in type I cells has remained elusive.

**Methods:**

Here we describe a development of a lung slice preparation that has allowed positive identification of alveolar type I cells within an intact and viable alveolar epithelium using living cell immunohistochemistry.

**Results:**

This technique has allowed, for the first time, single ion channels of identified alveolar type I cells to be recorded using the cell-attached configuration of the patch-clamp technique.

**Conclusion:**

This exciting new development should facilitate the ascription of function to alveolar type I cells and allow us to integrate this cell type into the general model of alveolar ion and fluid balance in health and disease.

## Background

Of fundamental importance to the optimisation of gas exchange in health and disease is the role of the alveolar epithelium in regulation of the liquid sub-phase in the postnatal lung. There is now unequivocal evidence to support both constitutive and stimulated Na^+^-driven, active vectorial transport of water from lung lumen to interstitium in the neonatal and adult lung (see [1-5] for reviews). During the final days of fetal development, steroid hormone-induced transcriptional upregulation of the amiloride-sensitive epithelial Na^+ ^channel (ENaC) ensures that, during labour, the huge surge in fetal adrenaline leads to channel opening and massive Na^+ ^flux out of the fetal lumen [6-9]; this drives osmotically-linked fluid reabsorption in preparation for the neonatal lung to take on the gas exchange role previously undertaken by the placenta. Maturation of the response appears to be under control of a number of environmental factors, most importantly oxygen [10-12]. Key to understanding this mechanism was the observation that transgenic mice which lack the α subunit of the ENaC channel complex die shortly after birth of alveolar flooding [13]. However, although there is a general consensus concerning the cellular and molecular basis of the fluid transport which clears the neonatal lung at birth, controversy still surrounds the issue of postnatal absorptive mechanisms where the role of ENaC in lung fluid homeostasis is less clear. Indeed, evidence is now emerging which supports a significant role for amiloride-insensitive pathways in adult lung fluid reabsorption. Of note are whole tissue studies in several different species [14-16]. Norlin *et al*. [15] demonstrated that up to 70% of basal guinea pig lung fluid reabsorption is amiloride-insensitive whilst in adult rat [16] and lamb [14], the amiloride-insensitive component is 55% and 30%, respectively. Junor and colleagues, employing the *in vivo *lamb lung preparation, demonstrated a pharmacology which was consistent with the involvement of cyclic nucleotide-gated cation (CNG) channels [14], a notion supported recently by Norlin *et al*. [16] who have shown direct upregulation of amiloride-insensitive absorption with cGMP. Expression of this phenotype appears to be under developmental control since pimozide (a CNG channel blocker) is ineffective in fetal lamb lung [17]. Recently, such *in vivo *observations have been corroborated in a cellular system by demonstrating directly, amiloride-insensitive, cGMP-evoked whole-cell Na^+ ^currents in postnatal alveolar epithelial cells [18].

Crucial information currently missing from the picture which defines alveolar fluid homeostasis concerns the potential differential location of key ion channel and transport proteins within the alveolar epithelium itself, *i.e *are they in alveolar type I cells or alveolar type II cells? Thus, until very recently, the processes which underlie physiological fluid reabsorption were believed to be physically situated exclusively in the alveolar type II cell. Although it is true that alveolar type II cells express the entire gamut of proteins believed to be sufficient for efficient vectorial ion, solute and water transport (see [19,20] for earliest reports of type II cell monolayer properties), the contribution to the reabsorptive response of alveolar type I cells has never been robustly investigated, due essentially to the difficulty in routinely isolating such cells. This problem has been confounded by the use of polarised monolayers of adult alveolar cells which have been consistently reported as being type II cultures but which by many parameters have characteristics of type I cells [21,22]. Several recent studies have suggested that type I cells are of importance to ion and water balance in the lung. Thus, employing immunohisto- and immunocytochemistry, two concurrent reports demonstrated expression of αENaC in both alveolar type I and type II cells [23,24], whilst aquaporin 5 was demonstrated to be exclusively localized to the apical membrane of the alveolar type I cell [23]. Using a relatively low purity cell preparation, alveolar type I cells became strongly implicated in ion transport by the demonstration that they transported Na^+ ^in an amiloride-sensitive manner; quantitatively, this sodium transport was almost 2.5-fold larger than that afforded by alveolar type II cells [24]. Evidence for the involvement of alveolar type I cells in fluid transport came from an *ex vivo *observation that the ouabain-sensitivity of the largest proportion of lung fluid reabsorption closely matched that of the α_2 _subunit of Na,K-ATPase (a subunit that was shown by those authors to be localized to type I cells [25]).

A further disadvantage of cellular studies on isolated alveolar epithelial cells is that they provide little information about the physiological interactions that occur between type I and type II cells. The fact that such interactions occur and are of physiological import has recently become apparent with the development of a co-culture system. This system has demonstrated that functional gap junctions exist between alveolar epithelial cells and that the expression profile of connexins that form these gap junctions differs depending on the cell type which is interacting [26,27].

Although type I cells are now implicated in alveolar ion transport, direct electrophysiological evidence of specific ion channels is thus far completely lacking. There are two main reasons for such a gap in our knowledge. Firstly, highly pure alveolar type I cell monolayers have not been generated on permeable supports (making short-circuit current measurements unfeasible). Secondly, isolated alveolar type I cells appear to be overly fragile and, as a consequence, have been unamenable thus far to patch-clamp studies. Neither of these problems may prove insurmountable with further advances in production of relatively pure alveolar type I monolayers [28], but the fact remains that no electrophysiological information from either cell type *in situ *is currently available.

In order to address directly the most important outstanding question in adult alveolar ion handling, *i.e*. what cells express which channels, we have developed a novel lung slice preparation which can be utilised for electrophysiological study. Using this technique we show that: a) cells within the alveolar epithelium remain viable for a number of hours; b) positive identification of living type I cells is routinely possible using imunocytochemistry and; c) single ion channels can be reliably recorded from the apical membrane of identified type I cells *in situ*. This advance will allow us to ascribe function to specific cell types within the alveolar epithelium and represents a novel methodology for investigating ion transport in fetal and postnatal lungs. Some of this work has appeared previously in abstract form [29,30].

## Methods

### Reagents

All compounds were of analytical grade and were purchased from BDH Laboratory Supplies (Poole, Dorset, U.K.) unless otherwise stated. Sodium isethionate, N-(2-Hydroxyethyl)piperazine-N'-(2-ethanesulfonic acid) 4-(2-Hydroxyethyl)piperazine-1-ethanesulfonic acid (HEPES), tetraethylammonium chloride (TEA), amiloride, niflumic acid and bovine serum albumin (BSA) were obtained from Sigma-Aldrich (Poole, Dorset, U.K.). Potassium isethionate was obtained from Fisher Scientific (Loughborough, Leicestershire, U.K.).

### Slice preparation

6–8 day old Wistar rats were killed in accordance with the Home Office guidelines. Heart and lungs were removed *en bloc*, and washed in a physiological solution containing (in mM): 140 NaCl, 5 KCl, 10 HEPES, 1 CaCl_2_, 1.2 MgCl_2_, 5 D-glucose, pH 7.4, 300 mOsM). Individual lung lobes were dissected and fixed directly to the jig of an Integraslice (7550 PSDS, Campden Instruments, Leicester, Leicestershire, UK) by cyanoacrylate adhesive. The Integraslice allows reproducible, thin slicing of delicate tissue without the need to fill with stabilizing agents such as low-melting point agarose; a manoeuvre which we have found to limit the ability of making patch-clamp recordings in living lung slices. 200 μm lung slices were sectioned on a transverse plane across the lobe. During sectioning, the temperature of the bathing physiological solution was maintained at 4°C using a temperature-controlled specimen bath (765 HP, Campden Instruments.). Lung tissue slices were then transferred to a 24-well plate containing ~2 ml of physiological solution and kept at 37°C in a humidified incubator gassed with 5% CO_2 _/ 95% air prior to immunohistochemical staining and electrophysiological recording.

### Live/Dead Staining

Lung tissue slices were treated with Live/dead^® ^viability/cytotoxicity assay kit for animal cells (Molecular Probes, Eugene, Oregon, USA) which was stored at -20°C and allowed to warm to room temperature prior to experimentation. The live (Acetoxymethylester of calcein, (calcein-AM), 4 mM) and dead (Ethidium homodimer (Eth-D), 12 mM) stock reagents were diluted to their final working concentrations (1 μM and 4 μM, respectively) in physiological solution (see above). The slices were then incubated in these solutions at room temperature for 30 minutes. Images were collected using BioRad CellMap confocal system (BioRad, Hertfordshire, UK) mounted on an Olympus BX50WI microscope (Olympus Optical Co. (Europa) GmbH, Hamburg, Germany). In order to confirm the validity of the live/dead staining, lung tissue slices were also treated with 1% Triton X-100 for 15 minutes prior to staining with 1 μM calcein and 4 μM EthD-1 solution for 30 minutes and subsequent imaging.

### Live immunostaining of alveolar type I cells

In order to identify positively living alveolar type I cells within the lung slice, the type I cell-specific, mouse monoclonal primary antibody, VIIIB2 [31], was employed. Lung slices were washed three times for five minutes with 2 ml of warmed PBS with Ca^2+ ^and Mg^2+ ^(Sigma-Aldrich, Poole, Dorset, U.K.). Lung slices were then incubated at 37°C for 1.5 hours in the presence of VIIIB2 (1:5 dilution). Lung slices were washed three times more for five minutes each in PBS and then incubated in a 5% solution of BSA for 15 minutes at 37°C, followed by washing three times for five minutes in warmed PBS. Slices were then labelled with aN anti-mouse IgG secondary antibody (Alexa Fluor 488, Molecular Probes, Strathclyde, Paisley, U.K.) at a 1:500 dilution and incubated for upwards of one hour at 37°C. Immediately prior to imaging, the lung slices were washed once again with warm PBS to remove any unbound antibody. Images were collected using BioRad CellMap confocal system mounted on an Olympus BX50WI microscope. In some experiments, lung slices were stained with either calcein-AM (1 μM, 30 minutes) or EthD-1 (4 μM, 30 minutes) following the usual VIIIB2 immunostaining protocol. In this cases, the secondary antibody was either a 1:500 dilution of mouse Alexa Fluor 546 secondary antibody (Molecular Probes) for calcein co-staining or a 1:500 dilution of mouse Alexa Fluor 488 for EthD-1 co-staining.

In a number of lung slices (from 5 animals), the mean number of dead cells per field (105 μM × 105 μM) was assessed (as EthD-1 positive nuclei). Such quantification was performed on untreated (n = 41), Triton-treated (n = 30) and VIIIB2-treated (n = 31) slices. Sample means were compared using one-way ANOVA followed by Bonferroni *post-hoc *test, with 0.05 taken as the level of significance.

### Electrophysiology

Lung slices were placed in a recording chamber mounted on the Olympus BX50WI microscope and alveolar type I cells were identified prior to patch clamp using standard fluorescence microscopy. Slices were held in place by a platinum ring with nylon cross wires in order to stabilize it for patch clamp. Recording pipettes were pulled from borosilicate glass and fire-polished (Narishige MF-83 microforge) and coated with Sigmacote^® ^(Sigma-Aldrich). Pipettes had resistances of 10-15MΩ when filled with pipette solution, which contained (in mM): 145 Na Isethionate, 10 HEPES, 1 CaCl_2_, 1.2 MgCl_2_, 5 D-Glucose, 10 mM TEA, 100 μM niflumic acid, 10 μM amiloride, with the pH adjusted to 7.4 with NaOH.

Following formation of a gigaohm seal, single-channel activity was recorded at room temperature in the cell-attached configuration of the patch-clamp technique. Cells were held at potentials ranging from +30 to -90 mV (Vm, assuming resting potential of -40 mV). Resistive-feedback voltage-clamp was achieved using an Axon mulitclamp 700A amplifier (Axon Instruments, Foster City, CA, USA). Voltage protocols were generated and currents recorded using pClamp 9.0 software, employing a Digidata 1322 A/D converter (Axon Instruments). Data were filtered (4-pole Bessel) at 2 kHz and digitized at 5 kHz. Data analyses were performed using the pClamp 9.0 suite of software (Axon Instruments).

## Results

### The lung slice preparation retains living cells

**Figure 1:**
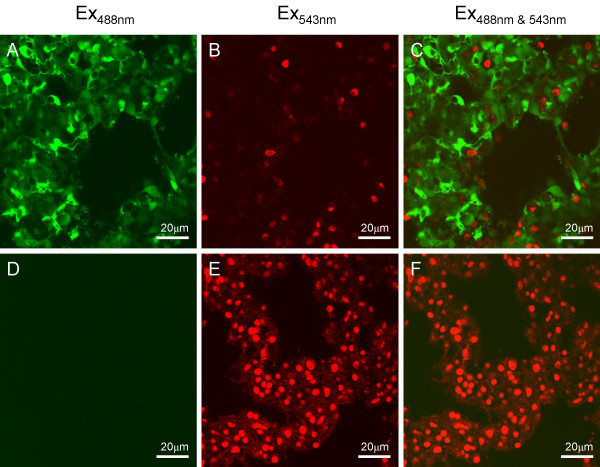
**Confocal images of rat lung slice co-stained with the LIVE/DEAD^® ^viability/cytotoxicity assay kit for animal cells. **A) A 200 μm rat lung slice loaded with 1 μM calcein-AM/4 μM EthD-1 and excited with light of 488 nm (green emission) to show viable cells. B) The same 200 μm rat lung slice loaded with 1 μM calcein-AM/4 μM EthD-1 and excited with light of 532 nm, (red emission) to show the potential dead cells. C) Overlay of images (A) and (B) showing that the majority of the cells are alive. D) A 200 μm rat lung slice loaded with 1 μM calcein-AM/4 μM EthD-1 following a 15 minute treatment with 1% Triton X-100 and then excited with light of 488 nm (green emission) to show viable cells. E) The same 200 μm rat lung slice loaded with 1 μM calcein-AM/4 μM EthD-1 following a 15 minute treatment with 1% Triton X-100 and then excited with light of 532 nm, (red emission) to show the potential dead cells. F) Overlay of images (D) and (E) showing that no live cells remain following treatment with Triton X-100.

**Figure 2:**
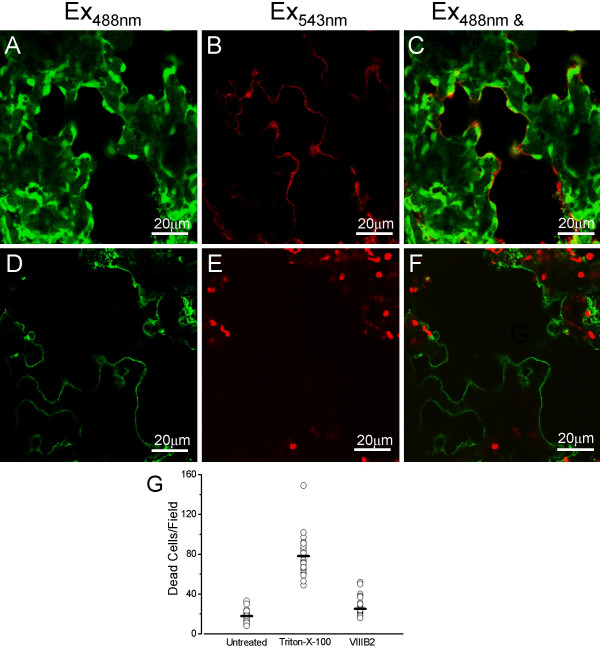
**Confocal images of rat lung slice co-stained with either the LIVE^® ^or DEAD^® ^viability stain and the specific alveolar type I cell antibody, VIIIB2**. A) A 200 μm rat lung slice incubated with the live stain (1 μM Calcein-AM) following live immunohistochemical staining with VIIIB2 primary antibody/Alexa Fluor 532 secondary antibody and excited at 488 nm (green emission) to show living cells. B) The same lung slice incubated with the live stain (1 μM Calcein-AM) following live immunohistochemical treatment with VIIIB2 primary antibody/Alexa Fluor 546 secondary antibody and excited at 532 nm (red emission) to show the Alexa Fluor 546 secondary antibody staining at alveolar type I cells. C) Overlay of images (A) and (B) showing that VIIIB2 immunoreactivity is clearly restricted to elongated, thin cells located at the edge of the alveolar space (indicated by the arrows), characteristic of ATI cells. D) A 200 μm rat lung slice incubated with the dead stain (4 μM EthD-1) following live immunohistochemical staining with VIIIB2 primary antibody/Alexa Fluor 488 secondary antibody and excited at 488 nm (green emission) to show secondary antibody staining of alveolar type I cells. E) The same lung slice incubated with the dead stain (4 μM EthD-1) following live immunohistochemical treatment with VIIIB2 primary antibody/Alexa Fluor 488 secondary antibody and excited at 532 nm (red emission) to show the EthD-1 positive, dead cell nuclei. F) Overlay of images (D) and (E) showing that VIIIB2 immunoreactivity and the dead cell stain do not co-localise. G) Graph showing quantification of dead cells per field (105 μM × 105 μM) following treatments indicated at the bottom of each data set.

The viability of cells within lung slices was assayed using the Live/Dead^® ^assay kit which differentially stains living or dead cells. Viable cells can be distinguished by the ability of their intracellular esterases to convert the non-fluorescent, cell-permeant calcein-AM ester to fluorescent calcein. Fluorescent calcein is retained within the cytoplasm of living cells and can be detected upon excitation at 488 nm (green emission). The damaged membranes of non-viable cells are permeable to EthD-1. Dead cells can be distinguished by nucleic acid-bound EthD-1 in their nuclei which can be detected upon excitation at 532 nm (red emission). The ability of this technique to distinguish between living and dead cells was confirmed using HEK 293 cells before and following treatment for 15 minutes with Triton-X-100 (data not shown). Following the successful optimization of the fluorescent live/dead assay in cultured cells, the same approach was utilized to determine the viability of the cells within the lung slices. As for the cell line, rat lung slices were loaded by incubation with 1 μM calcein and/or 4 μM EthD-1 in physiological solution for 30 minutes. Following this incubation, the majority of cells within a slice were positive for calcein (Figure 1A, C) whilst a small proportion of cells demonstrated EthD-1 positive nuclei (Figure 1B, C). The overlain images (Figure 1C) indicates that the majority of cells within the lung slice are viable. Pre-treatment with 1% Triton X-100 for 15 minutes resulted in a dramatic loss of calcein-positivity (Figure 1D, F) and a large increase in EthD-1 incorporation (Figure 1E, F). Quantification of the mean number of EthD-1 positive nuclei showed that Triton-X-100 treatment resulted in a significant, 4-fold increase in the number of dead cells per field from 18.0 ± 0.9 (n = 41) to 78.1 ± 3.3 (n = 30 slices; Figure 2G)). Thus, before such treatment, the majority of cells within the slice were alive whilst permeabilization with detergent resulted in cell death throughout the lung slice.

Utilizing the VIIIB2 antibody (to label selectively alveolar type I cells [31]) in conjunction with calcein fluorescence allowed estimation of the viability of specific cells within the epithelium. Figure 2A, B and 2C show a typical results from a 200 μm lung slice which was treated with 1 μM calcein-AM for 30 minutes following the VIIIB2 live cell immunohistochemical protocol. Again, the majority of cells were calcein-positive (Figure 2A). The mouse VIIIB2 immunoreactivity was clearly restricted to elongated, thin cells located at the edge of the alveolar space (Figure 2B), a pattern of immunoreactivity characteristic of alveolar type I cells [31]. The overlain images (Figure 2C) show the green cytoplasm of viable alveolar type I cells in close proximity to the VIIIB2 immunoreactivity at the plasma membrane. Furthermore, using the VIIIB2 antibody in conjunction with EthD-1 fluorescence allowed investigation and quantification of the effect of the immunohistochemical staining protocol of cell death within the slice. Figure 2D shows again, but this time using the Alexa 488 secondary antibody, that VIIIB2 staining is localised to the alveolar type I apical membrane. Figure 2E shows the modest cell death which has occurred as a consequence of the live immunohistochemistry and Figure 2F indicates that cell death is not typically induced in alveolar type I cells following the immnostaining protocol. The modest, but significant 1.4-fold increase cell death evoked by live immunohistochemistry is quantified in Figure 2G which shows that VIIIB2 treatment results in 25.3 ± 1.7 dead cells per field (n = 31) compared with 18.0 ± 0.9 in untreated slices. Excluding the VIIIB2 from the immunohistochemical protocol or replacing VIIIB2 with an antibody known only to bind an intracellular epitope (BKα-subunit antibody from Santa Cruz) resulted in no fluorescent signal (data not shown).

**Figure 3:**
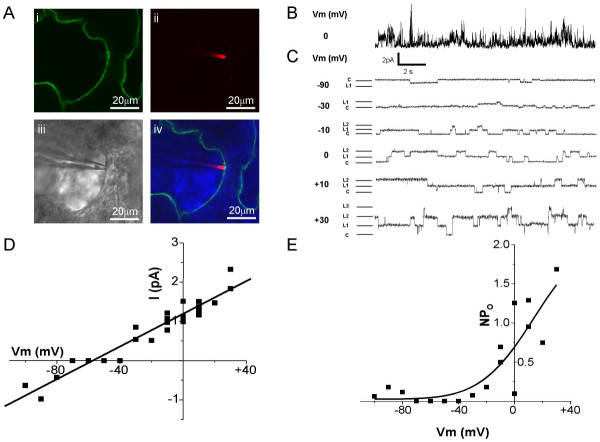
**Cell-attached single-channel recordings from identified alveolar epithelial type I cells in a rat lung slice. **A(i) A 200 μm rat lung slice immunostained stained with the mVIIIB2 antibody (Alexa Fluor 488 secondary antibody, green emission). (ii) Patch-clamp recording pipette in close proximity to an alveolar epithelial cells whose tip has been filled with TRITC (1:400). (iii) Transmitted light image. (iv) Overlay of images (i) and (ii) plus blue transmitted image to show that the patch recording pipette has formed a gigaohm seal on an VIIIB2-immunopositive alveolar type I cell. B) Exemplar current recording without 10 mM TEA in the pipette to demonstrate the complex nature of the current. Currents were recorded at 0 mV (Vm) and filtered at 2 kHz C) Representative family of cell-attached currents recorded from an alveolar epithelial type I cell, identified by live immunohistochemistry (as in (A)) in an acutely isolated rat lung slice. Single channel activity was recorded in the cell-attached configuration of the patch-clamp technique using a low chloride pipette solution with 10 μM amiloride, niflumic acid and 10 mM TEA. Currents were recorded at the potentials indicated to the left of each trace and were filtered at 200 Hz. L1, L2 and L3 indicate channel open levels 1, 2 and 3, respectively, whilst C indicates the channel closed level. D) Current voltage relationship of the single-channel activity recorded in 3 identified type I cells from 3 separate lung slice preparations. E) NPo (product of open state probability and number of channels) versus voltage plot of the single-channel activity shown in (E).

Having shown that labelled type I cells within the lung slices were viable, the patch clamp technique was utilized to examine ion channel activity in such positively identified cells. Figure 3Ai shows a 200 μm lung slice immunostained with VIIIB2 to identify alveolar type I cells. Again, VIIIB2 immunoreactivity was restricted to elongated cells located at the edge of the alveolar space, characteristic of type I cells. In order to show the position of the recording pipette, the tip was filled with tetramethylrhodamine isothiocyanate (TRITC, 1:400) as shown in Figure 3Aii. Figure 3Aiii shows light-field view of the pipette and tissue and the overlain images (plus the blue transmitted light image, Figure 3Aiv) indicates clearly that the tip of the patch pipette can be manipulated onto the apical membrane of an identified alveolar type I cell. Using this cell-attached configuration, single channel currents could be recorded routinely from VIIIB2-immunoreactive cells. Figure 2B shows an exemplar current trace recorded at 0 mV (Vm), filtered at 2 kHz and with 10 mM TEA excluded from the pipette solution. Such "unfiltered" currents are extremely complex in nature when the broad-spectrum potassium blocker, TEA, is excluded from the extracellular recording solution indicating that alveolar type I cells express at least two populations of K^+ ^channels – those which are TEA-sensitive and those which are not. However, when TEA was included in the pipette solution and the recordings were filtered at 200 Hz or lower, single channel openings could be resolved. A family of such currents recorded from an alveolar type I cell in the cell-attached configuration is shown in Figure 3C. Under the stringent recording conditions employed (where ENaC, the majority of potassium and all chloride channels are inhibited pharmacologically), changing the applied membrane potential from between -90 mV through +30 mV resulted in the activation of small conductance (Figure 3D), voltage-dependent channels (Figure 3E) whose currents reversed around -60 to -70 mV (Figure 3D), which is close to E_K _under the imposed ionic conditions. The current-voltage relationship (Figure 3D) of these channels recorded from three alveolar type I cells in separate lung slices gave a mean single channel conductance of 21 ± 3 pS. Figure 3E shows the product of the open state probability and the channel number (NPo) versus voltage relationship of the same channels and indicates that activity was voltage-dependent, activating at potentials more depolarized than -40 mV. These exemplar electrophysiological recordings show for the first time that ion channel activity can be successfully recorded from positively identified, living alveolar epithelial type I cells within an *in situ *lung preparation.

## Discussion

Until now, cellular studies on alveolar epithelial cells have been almost exclusively limited either to direct observation from freshly purified and cultured cell populations or by inference from *in vivo/ex vivo *measurements of lung function. Such limitations notwithstanding, an enormous amount of important information has been gathered and a widely accepted integrated model of the ion and fluid homoestatic processes which clear the lung of fluid at birth and keep it essentially dry thereafter (in both health and disease) has emerged. Thus, it is commonly believed that alveolar type II cells are the principal cellular element responsible for generating the transepithelial osmotic driving force and that alveolar type I cells represent relatively passive players in the vectorial transport process. This almost universally accepted belief is based on a number of key observations from many different researchers employing a wide variety of techniques. Although not an exhaustive list, such crucial data include the observations that: a) alveolar type II cells in culture express ENaC subunits (*e.g*. [32]); b) manipulation of culture conditions to emulate the alveolar air/liquid interface induces functional ENaC expression in type II cells [33]; c) ENaC is necessary for effective fluid reabsorption at birth [13] and contributes one component to the reabsorptive response required for the resolution of postnatal pulmonary oedema [34] and; d) β-adrenoreceptors, believed to be necessary for catecholamine-evoked fluid reabsorption are expressed in type II cells [35]. Whilst all these observations are undoubtedly true, there are two important reasons why a model of lung fluid homeostasis which is so centred on alveolar type II cells may not represent the entire picture *in vivo*. Firstly, alveolar type II cells rapidly transdifferentiate into a type I cell phenotype in culture (see [36] for recent review). Secondly, only a very limited number of studies have actually investigated the cellular properties of alveolar type I cells, either *in vivo *or *in vitro*. However, new information is beginning to emerge which suggests that type I cells have the potential to contribute significantly to overall alveolar fluid homeostasis. For example, they express β-adrenoceptors [37] and other important ion transport proteins [23,38], have the capacity to generate a large ouabain-sensitive Na^+ ^flux [25] and demonstrate significant amiloride-sensitive Na^+ ^uptake [24], and currents (Kemp, Kim and Borok, unpublished observations).

Clearly, one way to resolve the current controversies and to generate a model which includes data from both epithelial cell populations is to study cellular function of identified cell types *in situ*. To this end, an elegant *ex vivo *study has demonstrated Ca^2+ ^oscillations in alveolar type II cells which have been imaged in an isolated, perfused lung [39]. However, although this model has been used to puncture single alveoli with an injection microelectrode, it has not yet been amenable to single cell electrophysiology [40]. As a compromise, we report here the development of a technique which employs freshly cut lung slices to study the ion channel properties of identified alveolar type I cells *in situ*. The majority of cells within the lung slice are viable for up to 8 hours when bathed in physiological solution (Figure 1). The most important new aspect of the preparation is the ability to immunolocalize living alveolar type I cells within the slice. The most crucial tool for successful live immunostaining is a highly cell-specific antibody which is has been raised against an extracellular epitope; VIIIB2 is such an antibody and the data in Figure 2 show a pattern of live immunostaining which is unique to alveolar type I cells. The notion that this staining was cell specific was reinforced by the demonstration that employing a primary antibody known only to bind an intracellular epitope or removing the VIIIB2 from the protocol resulted in no fluorescent signal. A further important development was the adaptation of standard immunohistochemical techniques in order to reduce the amount of time that tissue is incubated with the various reagents. Thus, we optimised the staining protocols so that the entire procedure took less than three hours. This immunostaining was conducted entirely using physiological solutions and resulted in only a modest reduction in cell viability (see Figure 2). Crucially, this novel combination of lung slice and live cell immunohistochemistry has allowed us to identify viable alveolar type I cells *in situ *with sufficient accuracy to be able to manipulate patch-clamp electrodes onto their apical membranes. For the purposes of demonstrating the feasibility of the *in situ*, lung electrophysiological technique, we designed the pipette solution so that most ion channels within the membrane would be inhibited by employing an inhibitor cocktail which included TEA, niflumic acid, amiloride and low chloride. Under these conditions, we have shown that single channel currents can be recorded (Figure 3C) with a resolution sufficient to undertake basic biophysical characterization. Interestingly, employing a pipette solution which did not contain TEA resulted in recordings which were not readily amenable to robust analyses. Clearly, the challenge will now be to dissect each individual channel using a combination of pharmacology and molecular abrogation technologies in excised patches, manoeuvres outwith the scope of the current feasibility study. However, reducing the number of channel types within a cell-attached patch by employing a high concentration of TEA has allowed us to show that alveolar type I cells express a 23pS, voltage-activated conductance. The analyses of these channels shown in Figure 3 indicates that this channel is primarily permeable to potassium although a contribution from other ions cannot be completely rules out (Figure 3D). Further, this channel is TEA-insensitive and shows voltage-activation in the physiological range (Figure 3E). These characteristics suggest that the most likely potassium channel candidate underlying the recorded cell attached patch current belongs to Kv family, perhaps Kv1.7 (see [41] for review of known potassium channel characteristics). Potassium channels have also been reported in alveolar type II cells [42,43] where they are proposed to contribute to the regulation of driving forces across the epithelium. A parallel function may also hold true for such channels expressed in type I cells.

Finally, in addition to the feasibility of recording single ion channels expressed in alveolar type I cells, this technique has potentially much wider application and should, for example, allow investigation of Ca^2+ ^and pH homeostasis using fluorescent indicator systems in individual cell populations and to study the interactions between alveolar type I and II cells *in situ*.

## Conclusion

We report a development of the lung slice technique which allows immunolocalization of living type I cells within the alveolar epithelium. Using this novel positive identification, we have described a method which allows, for the first time, patch clamp analysis of single ion channels expressed on the apical membrane of alveolar epithelial type I cells *in situ*.

## Authors' contributions

All authors contributed to the conception and design of the study and to the discussion of the data. SB developed the lung slicing and immunostaining protocols. SB, ZB and PJK developed the live staining to identify type I cells whilst SB, HSM and PJK developed the *in situ *electrophysiology. The initial manuscript was written by HSM, SB and PJK and the final editing was conducted jointly by all the authors.
